# Optimization of Enzymatic Deproteination of Northern Shrimp (*Pandalus borealis*) Shell Chitin Using Commercial Proteases

**DOI:** 10.3390/md22100445

**Published:** 2024-09-28

**Authors:** Julia Pohling, Vegneshwaran Vasudevan Ramakrishnan, Abul Hossain, Sheila Trenholm, Deepika Dave

**Affiliations:** 1 Marine Institute, Center for Aquaculture and Seafood Development, Memorial University of Newfoundland, St. John’s, NL A1C5R3, Canada; julia.pohling@mi.mun.ca (J.P.); vvasudevanra@mun.ca (V.V.R.); sheila.trenholm@mi.mun.ca (S.T.); 2 Department of Biochemistry, Memorial University of Newfoundland, St. John’s, NL A1C 5S7, Canada; abulh@mun.ca; 3 Faculty of Land and Food Systems, The University of British Columbia, Vancouver, BC V6T 1Z4, Canada

**Keywords:** high-quality chitin, commercial proteases, Atlantic shrimp, optimization, waste utilization

## Abstract

Shrimp shells are a key source of chitin, commonly extracted through chemical methods, which may cause minor molecular damage. Nowadays, there is great interest in achieving close to zero protein content in crude chitin in order to use it for high-end markets. Therefore, this study optimized the enzymatic deproteination using two commercial proteases (SEB Pro FL100 and Sea-B Zyme L200) for effective and fast removal of residual protein from Northern shrimp (*Pandalus borealis*) shell chitin for the first time. The protein content was determined using both the Kjeldahl method and amino acid analysis using gas chromatography–mass spectrometry (GC-MS). The performance of papain (Sea B Zyme L200) was superior to fungal protease (SEB Pro FL100) for this application, and it achieved residual protein content of 2.01%, while the calculated optimum for the latter enzyme was 6.18%. A model was developed using 2^4^ factorial design, and it was predicted that the lowest residual protein content using fungal protease and papain could be achieved at the following conditions: a pH of 4.2 and 7, and an enzyme concentration of 4 and 1.5%, respectively. Thus, the low-protein content obtained using enzymatic deproteination could be an alternative approach to the traditional methods, indicating their potential to produce premium-quality chitin.

## 1. Introduction

Chitin is a natural polysaccharide that is abundantly found in the exoskeleton of arthropods (crustaceans and insects) and fungi. Chitin and its deacetylated derivative, chitosan, have a versatile range of applications in the medical, environmental, cosmetic, and food/nutrition industries [1,2]. Typical for a natural polymer, chitin’s molecular structure and macrostructural arrangement in the cuticle vary widely between sources, seasons, body parts, animal health, and extraction methods [3]. The most critical descriptors of chitin are the degree of deacetylation (DDA) and the molecular weight, which render chitin and chitosan distinct properties [4,5,6]. Inconsistencies between chitosan products are considered one of the main reasons why chitosan is not yet approved as a medical ingredient and a generally recognized as safe (GRAS) substance, although it is often claimed to be a GRAS [7,8]. In contrast to synthetic polymers, parameters concerning chitin synthesis are completely out of our control, while parameters during shell handling and chitin extraction are somewhat controllable.

Northern shrimp (*Pandalus borealis*) is a small, soft-shelled species, and plays it a vital role in the Newfoundland and Labrador (NL) fishing industry [9]. According to Fisheries and Oceans Canada (DFO), in 2021, the total landing volume of shrimp in NL was 35,998 t (29.7% of the total shellfish landing), with a landing value of $140 million [10]. In NL, *Pandalus borealis* is machine-peeled in fully automated processing facilities. The shell, amounting up to 75% of the harvested biomass, is landfilled, composted, disposed at sea, or used in low-value applications, such as fertilizer. The shell is composed of approximately 17% chitin, 41.9% protein, 34.2% minerals, and 5.2% lipids [11]. While the value of chitin in the shells has been recognized in Canada for years, industries have been struggling to establish economically viable businesses. Factors such as production cost, health and safety regulations around the transportation, storage, and handling of large volumes of concentrated acids and bases, as well as wastewater treatment, are the major challenges to recovering chitin from shrimp.

Chitin can be extracted by several chemical, enzymatic, or biotechnological methods to remove mineral deposits, fats, proteins, and pigments [4,6,7]. The majority of the world’s chitin is produced using a traditional chemical approach. Minerals (mainly calcium carbonate) are dissolved by reaction with hydrochloric acid. Proteins are denatured and washed off using a dilute sodium hydroxide solution at elevated temperatures (below boiling). While this process is very reliable and consistently produces chitin of high purity, some minor molecular damage, mainly in the form of depolymerization, occurs [5]. In recent decades, the focus of the research has shifted away from chemically-intensive processes, and towards developing gentle extraction methods that minimize changes to the native molecular structure [7,8]. For example, demineralization has been achieved successfully using citric and acetic acids [12,13,14], as well as microwave-assisted deep eutectic solvent (DES) extraction [15]. The green approach for removing proteins from chitin replaces sodium hydroxide with proteolytic enzymes [4,6,11]. Despite the research efforts, caustic deproteination using sodium hydroxide is still the method of choice for protein removal because the process is simple and removes protein reliably and completely [4,5,6,15]. However, the agreement on which alternative process is best is still ambiguous. The advantage of enzymatic protein removal is that it allows for the recovery of the shrimp protein hydrolysate as a secondary product. Shrimp protein is particularly attractive as it contains all of the essential amino acids required by the body. The residual protein content in enzymatically deproteinated chitin has been a major issue in preventing the widespread commercialization of green processes. Achieving a high-purity chitin is vital for several reasons. Because natural polymers like chitin or cellulose compete with highly pure synthetic polymers for many applications, it is inherently essential to remove all other contaminants in order to achieve food-grade or biomedical-grade products, and to ensure that the polymer can be further processed without the presence of interfering substances [7,8].

While protein and ash contents of <1% are acceptable, the lower the residual contaminants, the higher the potential suitability for high-end markets [16]. The desire is to achieve a protein content as close to zero as possible. From the medical point of view, protein content should be minimized to avoid allergic reactions, which can occur as an immunological response to protein-based allergens. The presence of allergens compromises their clinical usage [16]. In particular, the protein tropomyosin has been identified as a potent allergen that should be removed completely [17]. From the processing point of view, a low residual protein content in chitin improves the conversion process to chitosan. In this process, called deacetylation, chitin is converted to chitosan under harsh alkaline conditions (>50% NaOH) at high temperatures (>100 °C). The reaction produces chitosan with a DDA of around 80–90%. To achieve a higher DDA, the process must be repeated several times. The higher-purity chitin achieves a higher DDA in the first step of conversion, shortening the deacetylation process and the number of times the product is exposed to harsh denaturing conditions. Because each deacetylation step causes some depolymerization, high purity of chitin is critical in producing high molecular weight chitosan [18]. Therefore, this study focused on optimizing enzymatic deproteination using two commercial proteases for effective and fast removal of residual proteins from Northern shrimp (*Pandalus borealis*) shells to produce chitin for the first time. This approach could address the full utilization of Northern shrimp by utilizing processing waste and producing high-quality and low-protein chitin.

## 2. Results and Discussions

### 2.1. Enzymatic Deproteination

Optimization of enzymatic deproteination was performed for two industrial enzymes, SEB Pro FL100 and Sea B Zyme L200. The performance of Sea B Zyme L200 was superior to SEB Pro FL100 for this application, and it achieved a residual protein content of 2.01%, while the calculated optimum for SEB Pro FL100 was 6.18%. In the following two sections, the detailed results of optimization experiments are presented.

### 2.2. SEB Pro FL100

Table 1 shows the residual protein content for the 2^4^ factorial design. The model predicts the theoretical residual protein content of 3.75% at the following conditions: pH 3; temperature, 55 °C; enzyme concentration, 2.58%; and reaction time, 2 h. The deproteination reaction appears to be completed within the first 2 h. When the conditions were favourable, the enzyme was able to produce a residual protein content between 5–8% within 2 h. However, when they were unfavourable for deproteination, the residual protein content ranged from 11–16%, even after a 12 h reaction. None of the parameter combinations tested within the matrix were able to achieve protein contents below 5%.

**Figure 1:**
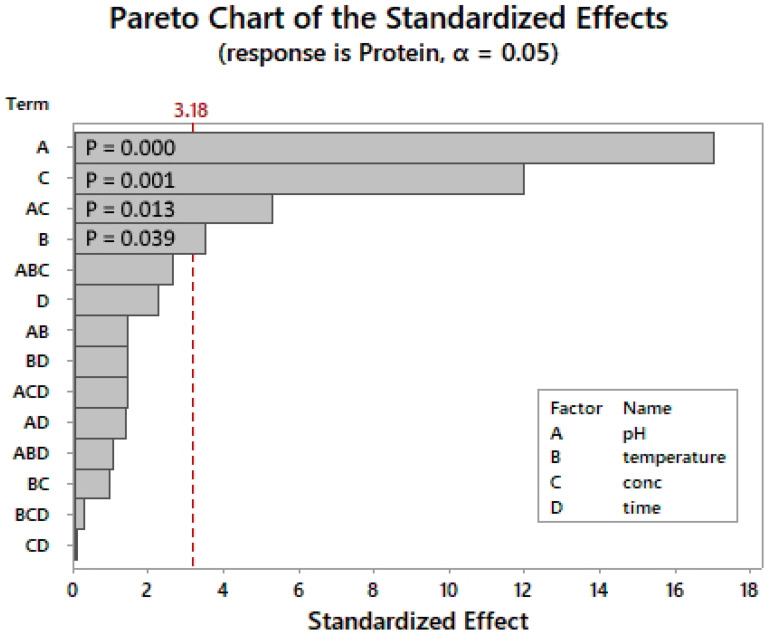
SEB Pro FL100: Pareto chart of standardized effects (2^4^ factorial design)*. p*-value is the probability used to assess the statistical significance of the result.

As shown in the Pareto chart (Figure 1), the most important individual factors are the pH, enzyme concentration, and temperature. The pH × enzyme concentration was the only significant two-way interaction identified with a *p*-value of 0.013. The reaction time had no significant influence on the response factor.

**Figure 2:**
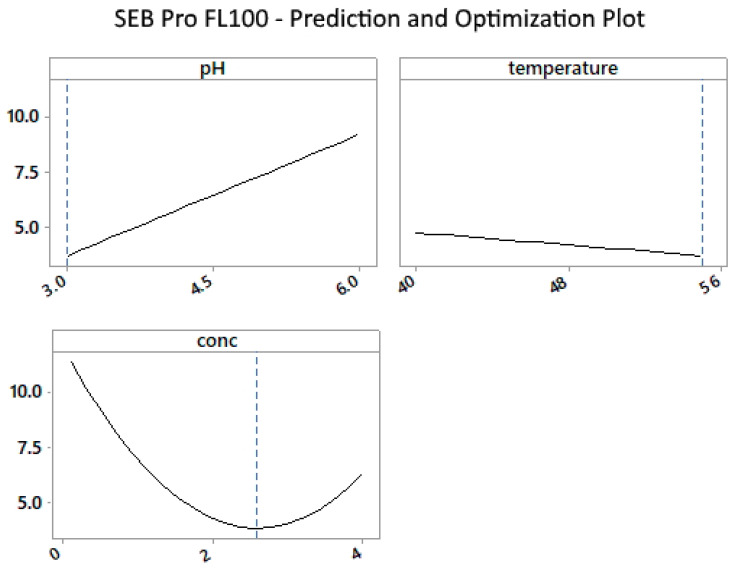
SEB Pro FL100: Prediction and optimization plot for mean of protein vs. pH, temperature, and enzyme concentration.

Figure 2 shows that the enzyme works best at the lower end of the pH range and drastically decreases as the pH increases. Enzyme concentrations above 2% were required to lower residual protein levels to below 5%. The effects of temperature were less pronounced, but slightly less protein residue was observed at the upper-temperature limit, supporting the findings in Figure 1.

**Figure 3:**
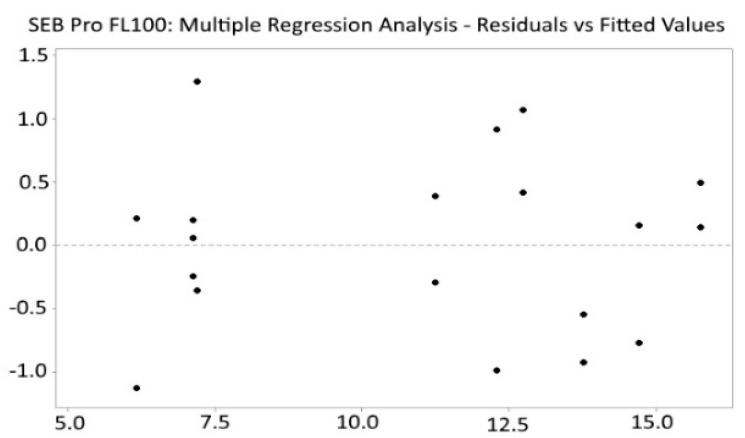
SEB Pro FL100: Residuals vs. fitted values.

The residual plot of multiple regression analysis was randomly distributed (Figure 3), indicating that the model is unbiased (R^2^ = 99.54).

Based on the data obtained, the levels for reaction time were chosen too broadly, and they did not correctly bracket the early phase of deproteination (<2 h), during which the dynamics of deproteination would be best-observed. The pH and enzyme concentration strongly influenced the reaction efficiency, and the levels chosen to study their impact were too wide. To further narrow down the optimal pH, reaction time, and enzyme concentration, a factorial design was created using tighter levels, as described in the materials and methods section. Ten additional experiments were selected from a full factorial design investigating the factors of time, pH, and enzyme concentration only, bracketing the optimum more narrowly than the initial 2^4^ factorial design (Table 2).

**Figure 4:**
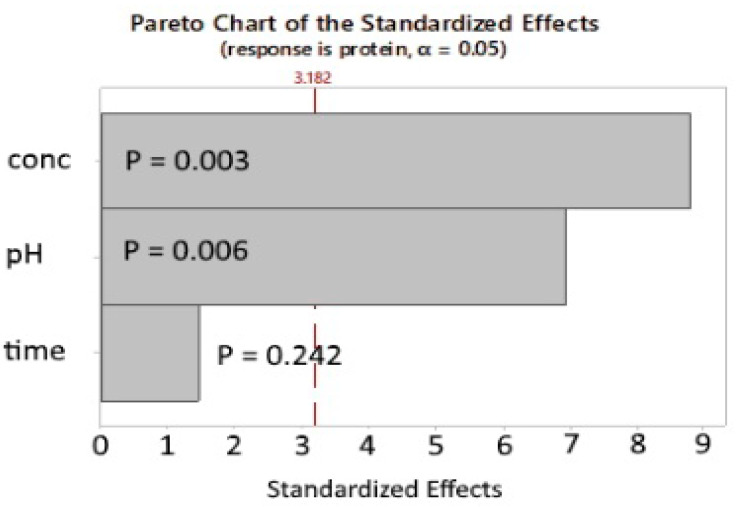
SEB Pro FL100: Additional experiments, Pareto chart of the standardized effects. *p*-value is the probability used to assess the statistical significance of the result.

The model predicted that the lowest residual protein using SEB Pro FL100 could be achieved at pH 4.2 and an enzyme concentration of 4%. At these conditions, the predicted residual protein content is 6.18%. The Pareto chart of the standardized effects (Figure 4) confirms the results of the initial factorial design, where the enzyme concentration and the pH are the most important factors, as indicated by the low P-value of 0.006 and 0.003, respectively. Furthermore, the reaction time is not statistically significant, indicating that the reaction occurs rapidly within the first hour. Due to the small sample size, the validity of the *p*-value should be interpreted with caution. Marzieh et al. [19] used the proteases Trypsin (pH 7.7) and Ficin (pH 7.5) to deproteinate *Litopennaeus vannamei* shells at room temperature for 1–4 h, using varying enzyme concentrations ranging from 0.1–0.033%. Sodium metabisulfite was used as an additive because of its ability to reduce disulfide bonds, increasing protein solubility. Agreeing with our study, the reaction time was found to be relevant only in the first 2 h. The lowest residual protein content was 5.31% for Trypsin and 6.1% for Ficin.

**Figure 5:**
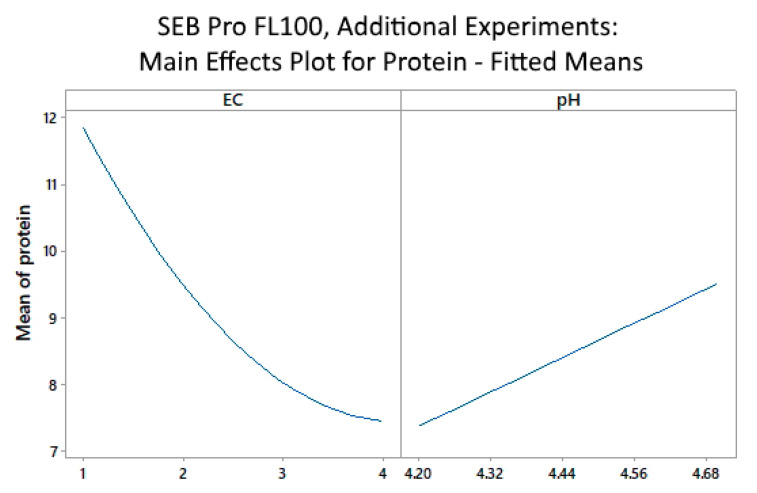
SEB Pro FL100: Additional experiments, main effects plot for mean of protein vs. enzyme concentration (EC) and pH.

The main effects plot (Figure 5) suggests that even higher enzyme concentrations in the range of 4 vol% may be needed to achieve optimal protein removal, confirming earlier results that a lower pH improved protein removal.

**Figure 6:**
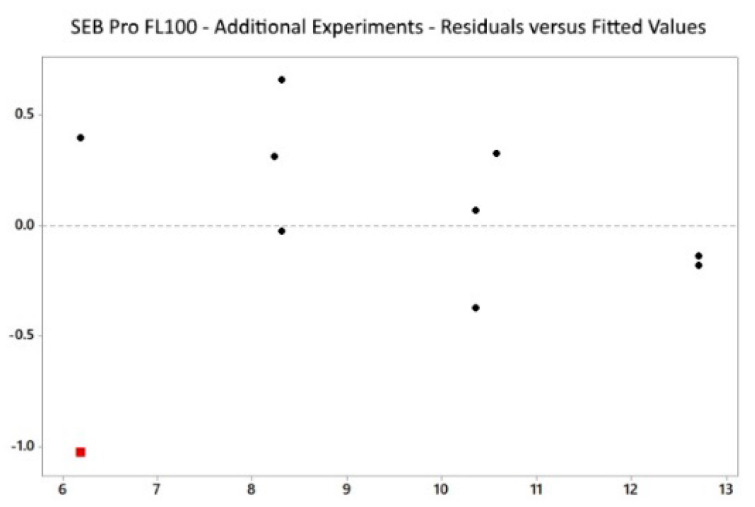
SEB Pro FL100: Multifactorial design, residuals vs. fitted.

The residual vs. fitted plot (Figure 6) showed randomly distributed values, indicating that our model was non-biased. However, one outlier was detected for experiment ID 1 at 5.15% residual protein, where the result for residual protein was significantly lower than the predicted value. Moreover, Table 3 lists the five best alternative solutions for SEB Pro FL100, producing theoretical residual protein contents between 8–13%.

### 2.3. Sea-B-Zyme L200

Table 4 shows the residual protein content for the 2^4^ factorial design. Based on the results, the theoretical residual protein content after treatment with the papain-based commercial protease is 2.3% at the following optimized conditions: pH 7; temperature, 70 °C; enzyme concentration, 1.5%; and reaction time, 2 h. Table 5 shows the top five alternative solutions.

**Figure 7:**
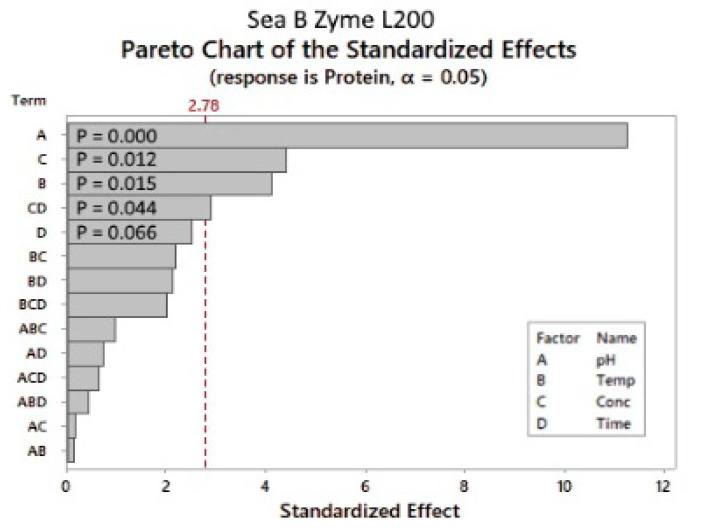
Sea-B-Zyme L200: Pareto chart of the standardized effects. *p*-value is the probability used to assess the statistical significance of the result.

As shown in Figure 7, the parameter with the most significant effect was the pH, followed by the enzyme concentration and reaction temperature. The reaction time had an insignificant influence on the response factor within the tested limits (Figure 7). The only significant two-way interaction was the enzyme concentration × reaction time, with a *p*-value of 0.044.

**Figure 8:**
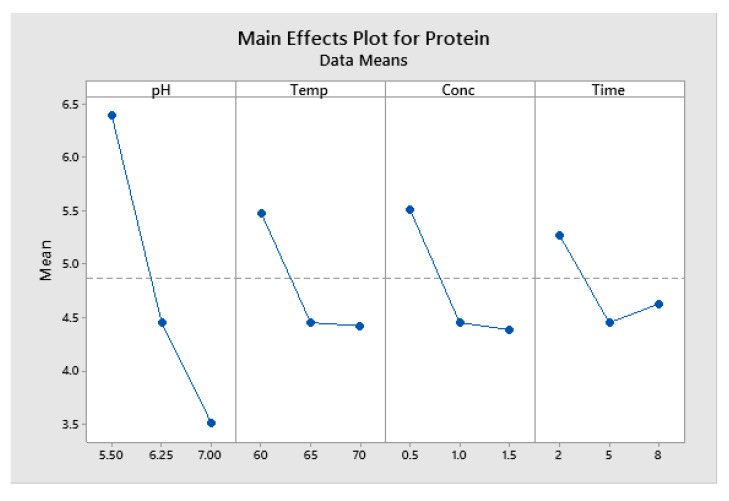
Enzymatic deproteination with Sea B Zyme L200: Main effects plot for protein (data means).

The main effects plot (Figure 8) results indicate that increases in the pH result in a sharp drop in the residual protein content. Temperature, concentration, and time also saw sharp decreases between low and centre points, but only minimal further reduction between centre points and upper levels.

**Figure 9:**
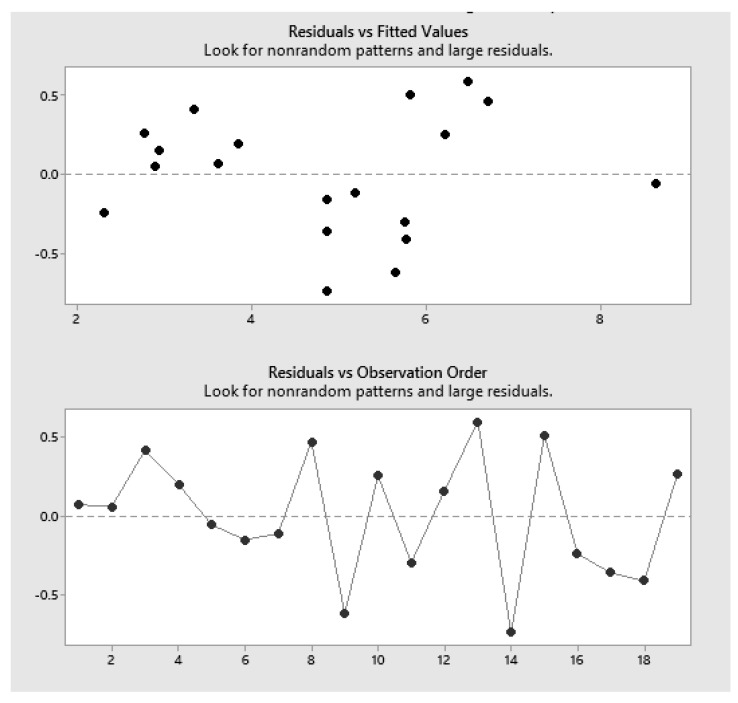
Sea-B-Zyme L200: Residuals vs. fitted values.

The residuals plot shows some clustering and is not randomly scattered around zero, indicating that our model is not completely unbiased (Figure 9). At various places along the predicted vs. actual plot, the model over- or under-predicts the response, despite the high R^2^ value of 99.65. Values have a normal distribution, and no unusual data points were detected.

### 2.4. Confirmation Runs, Sea B Zyme L200

Confirmation runs were performed for Sea B Zyme only, due to its superior results over SEB Pro FL100. Twenty confirmation runs were performed at the optimal predicted conditions of 1.5% enzyme concentration, 70 °C reaction temperature, and 2 h reaction time. All confirmation experiments were performed using the same shell material that was used for optimization experiments. The residual protein content was determined using both the Kjeldahl nitrogen estimation and the amino acid analysis using GC-MS. All tests were performed in duplicate, and the results are shown in Table 6.

The average residual protein content was 1.65 and 2.01%, as determined by the Kjeldahl method and GC-MS analysis, respectively. This was slightly lower than the value of 2.3% residual protein that was predicted by the 2^4^ factorial design. The Kjeldahl approach had a slightly higher standard deviation than amino analysis, indicating that the latter may have higher precision. Overall, the results of the two methods are in agreement. The quantification of residual protein in chitin is not yet standardized, and research groups use several different methods for evaluating deproteination success. One of the most common approaches described in the literature is the estimation via the Kjeldahl method, which we also used in this study. This approach relies on the assumption that the chemically deproteinated chitin reference is perfectly pure, yet fully acetylated. This is an inherent source of error, as intensive deproteination conditions also cause partial deacetylation, and small amounts of residual protein may still be present. Amino acid analysis via GC-MS is considered the most precise method for the determination of protein content in chitin, since it directly measures amino acid concentration, and is especially useful when determining very small amounts of residual protein residue in chitin, where precision is relevant [20]. We, therefore, double-tested the samples of the confirmation run with both methods and compared the results. There was a very good alignment between values obtained from the GC-MS amino analysis and the Kjeldahl estimation. Our results show that the Kjeldahl method, as described here, is a reliable method to quickly estimate the residual protein content and identify optimal deproteination conditions.

In terms of enzymes, papain was able to produce higher-purity chitin at a lower enzyme concentration and, therefore, provided the most efficient and economical deproteination solution. Both papain and fungal proteases have previously been reported for use in chitin deproteination. Earlier studies described the deproteination of chitin at pH 5.6–6.0 and 37.5 °C for 60 h [21]. Boonkam et al. [22] reported optimal conditions for papain-catalyzed deproteination at pH 8 and 40 °C for 1 h, using 1 unit of papain/ g of shrimp. Rohyami et al. [23] had a different research focus and investigated papain’s ability to cause partial deacetylation of chitin. In their study, shrimp shells were deproteinated with papain at concentrations ranging from 0–25%. The best results were achieved at 25% papain, which could result in a DDA of 41%. Valdez-Pena et al. [24] screened several industrial proteases for deproteination efficiency in shrimp heads (*Litopennaeus vannamei*) before demineralization, and achieved the lowest levels of residual protein, at 2.75%, using Alcalase, and between 3–4% for papain and a fungal protease from *Aspergillus oryzae*. Moreover, Deng et al. [25] used a two-step enzymatic treatment to deproteinate *Litopenaeus vannamei* shells. They tested aspartic protease P6281, saccharopepsin, Alcalase, and papain, and found that P6281 and saccharopepsin produced the best deproteination results. The degree of deproteination for papain was only 42.3% compared to 71.2% for protease P6281. Residual protein content was determined by incubating the shrimp shell sample in 1 M NaOH at 40 °C for 3 h, and then quantifying the protein content of the supernatant using the Kjeldahl approach. The degree of deproteination was calculated from the Kjeldahl result, and the observed weight reduction was calculated during deproteination. 

On the other hand, Gagne and Simpson [26] used chymotrypsin and papain to deproteinate shrimp waste obtained from Eastern Quebec, Canada. They achieved the lowest residual protein contents of 1.8% using chymotrypsin and 2.8% using papain. The papain reaction was conducted at 38 °C and pH 8.7, with an enzyme/waste ratio of 10:1000. While the reaction was run at a much lower temperature, the final protein residue was comparable to our study. Vazquez et al. [27] deproteinated squid pens (*Illex argentines*) using a NaOH-based process, as well as an enzymatic approach using the three industrial proteases, namely Alcalase, Esperase, and Neutrase. After deproteination, both solid and liquid fractions were analyzed. Protein in the chitin sample was calculated as the sum of amino acids analyzed by the ninhydrin method using an amino acid analyzer. Protein in the liquid fraction was determined using the Lowry assay. The results showed that Esperase was the best enzyme for the deproteination of squid pens, followed by Alcalase and Neutrase. Dhanabalan et al. [28] investigated a combination of enzymatic and chemical deproteination of Acetes shell, a tiny, soft, shrimp-like Arctic krill. Controlled enzymatic deproteination with Alcalase at an enzyme/substrate ratio of 1.53 at 53.5 °C for various lengths of time was performed until the specified degree of hydrolysis was reached (5, 15, and 30%). This was followed by a mild deproteination in NaOH to lower the residual protein to approximately 1%. These samples were compared to chitin which was deproteinated in 3% NaOH. The deproteination success was assessed using the Kjeldahl approach, like the current study. The lowest residual protein content achieved enzymatically was 3.33%. The authors note that, unlike the enzymatic process, the secondary NaOH treatment could remove a large portion of lipophilic pigments. Enzymatically deproteinated chitin has a higher degree of acetylation than chemically extracted chitin.

In addition, Younes et al. [29] investigated five different crude protease extracts for the deproteination of shrimp shells (*Metapenaeus monoceros*). The best results were achieved with protease from *Bacillus mojavensis* (A21), with a residual protein content that was 5.8% higher than the chemically deproteinated control sample, as determined by Kjeldahl analysis. The performance of the crude enzyme preparation was also found to be superior to the control enzyme bromelain, and Alcalase. In addition, six microbial and nine fish alkaline crude proteases were used in deproteinating shrimp shells (*Metapenaeus monoceros*). The best results were achieved for *Bacillus mojavensis* (A21) and *Balistes capriscus* (grey triggerfish) proteases with 77 ± 3 and 78 ± 2%, respectively. The degree of deproteination was calculated from the Kjeldahl crude protein content of the solid sample and the observed weight reduction during deproteination [30,31].

The studies above demonstrate that fast protein removal from chitin within a few hours is possible with several proteases. No protease clearly stands out, but Alcalase has been used in many studies and has become a common enzyme used as a performance reference. The studies have in common that there is always low residual protein content after treatment, ranging from 1.8–6.1% in most studies. The results of papain deproteination presented in this study align well with the most successful deproteination studies. The protein residue found in enzymatically deproteinated chitin has been attributed to covalent bonds between protein and chitin, hydrogen bonds, and hydrophilic interactions. Lastly, spatial restrictions limiting access to the substrate inside the chitin network are thought to contribute to incomplete deproteination [4,6,15,32,33].

### 2.5. Calculation of Total Deproteination during Chitin Extraction

**Figure 10:**
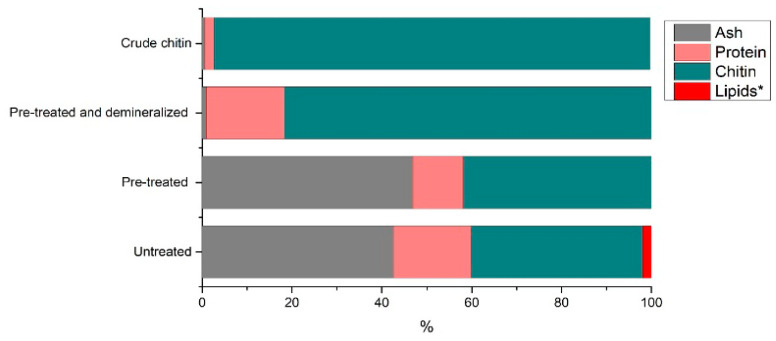
Compositional changes throughout chitin extraction (assuming lipid concentration is 0% and * indicates unpublished data).

Throughout the chitin extraction process, protein is removed step by step. While the deproteination step focuses on removing protein bound to shells, some protein is removed prior to the deproteination step by simply grinding and washing the shells. This protein includes meat protein particles, contents of the cephalothorax, and protein loosely attached to the outside of the shell. Figure 10 displays the proximate composition of the shrimp shells throughout the chitin extraction process. 

The chart compares the main shell constituents, protein, minerals, lipids, and chitin on a dry basis. In earlier studies on processing shrimp shells using the same pre-treatment and demineralization process (unpublished), we found lipid content of 1.99% in untreated shells, and once pre-treated, the lipid content was undetectable by the Soxhlet extraction. This finding confirmed published values for lipids in shrimp by-products, ranging from 2–3.15% in untreated *Penaeus kerathurus* and *Metapenaeus monoceros* shells [34] to 0.3–0.5% in *Pandalus borealis* [35]. Lipid quantification of the deproteinated sample was therefore not performed, and we assumed a value of 2% for raw shrimp shells and zero for all subsequent process samples to visualize the general composition. The residual protein content determined via GC-MS, which was slightly higher than the Kjeldahl value, is used for these calculations to present the worst-case scenario. Pre-treatment removes the loosely attached proteins. Demineralization removes minerals, leaving a very small amount of residual ash. Finally, deproteination reduces the residual protein content to 2.01% (the average residual protein content from the 20 confirmation runs). A final chitin purity of 97.12% is achieved.

Chitin purity depends not only on the deproteination process itself, but also on several other factors. In our study, we wanted to create ideal conditions for deproteination, not only by optimizing the deproteination step, but also by utilizing pre-treated, suitable raw materials. For example, the shell material used in our study was of exceptional freshness and was processed immediately after collection from the processing plant without a drying step or interim storage. The small particle size provided a high surface area to allow the enzyme to access the proteins. Moreover, demineralization was performed prior to deproteination, as recommended by Younes et al. [6], to increase the accessibility of the protein embedded in the chitin network. Finally, *Pandalus borealis* is a thin-shelled crustacean species, and among the smaller species harvested of food. Thin shells are generally easier to process for chitin extraction due to smaller mineral deposits and better accessibility of the protein to chemicals and enzymes [6,36]. To determine the economic viability of enzymatic chitin purification processes, a biorefinery approach is necessary, which assesses not only the cost of the enzymatic deproteination step, but the entire chitin extraction process, including the potential additional revenue from a protein hydrolysate product and the cost savings for energy and chemical handling safety measures. Testing of processes on pilot-scale processing is essential to obtain such feasibility assessments, but available data are minimal [37]. Most of the literature on chitin extraction cites insufficient chitin purity, high enzyme cost, and long reaction times as the main reasons why enzymatic deproteination processes have not been widely implemented on an industrial scale [5].

Some researchers have addressed purity issues by applying a short, gentle caustic treatment following enzymatic deproteination. For many applications, a residual protein content of <5% is acceptable (industrial and some food applications). The low residual protein content may not be critical if used as raw material for chitosan production, as it is removed during caustic deacetylation. The development of internationally accepted chitin purity standards would help in determining what level of purity is required. The high cost of enzymes has been addressed in different ways. Some authors use industrial enzymes, while others use crude protease, immobilized protease, or ferment chitin with protease-producing microorganisms [5,38]. Even still, an enzymatic approach is almost always more expensive or time-consuming compared to the chemical process using NaOH and HCl. Some of this higher cost can be offset by creating secondary products from the protein hydrolysate [25], especially in the age of society recognizing the need for high-quality protein sources, and the technical capability of turning a hydrolysate into useful and appetizing forms (e.g., peptides). Shrimp protein is particularly attractive because it contains not only protein but also pigment astaxanthin, one of the strongest natural antioxidants, which is responsible for the colour of egg yolks and salmon fillets. This potential is lost in the chemical process, where protein is destroyed. Enzymatic deproteination of chitin with commercial proteases takes as long as the average caustic deproteination reaction, roughly 2 h [5]. Significantly longer reaction times of up to several days are only experienced when chitin is fermented with lactic acid and protease-producing bacterial strains. Lastly, the local production setting must be considered when evaluating the cost-effectiveness of a chitin extraction process. Strict regulations can quickly make a process economically unfeasible, although reagents may be cheap. For example, in some countries with strict environmental laws and remote shrimp processing locations, the cost of establishing the necessary infrastructure to permit a chemical process, such as wastewater treatment, freshwater usage, worker health and safety regulations, and emergency first response capabilities, will quickly outweigh the economic benefit. Enzymatic processes are significantly more attractive for implementation in remote areas of industrialized countries. While materials are more expensive, enzymatic processes are performed in mild and non-hazardous conditions. Waste management is significantly easier, and bulk chemical transportation and storage are eliminated. However, to compete with highly pure, chemically extracted chitin, the processes must be highly optimized and find a niche consumer market.

Overall, the optimization of reaction parameters resulted in chitin with theoretical residual protein contents of 6.18 and 2.3% for fungal protease and papain, respectively. Papain performance was verified in twenty confirmation runs and produced an average residual protein content of 2.01%, outperforming the theoretical results of the factorial design. The two enzymes were selected based on different characteristics. Papain was selected for its advertised performance of producing non-bitter hydrolysates from seafood. The enzyme has a declared activity of 5500 PU/mg, and has activity over a wide pH range, from 4–10, with the optimum somewhere between 5–8 [39,40]. A drawback of papain was the high-temperature optimum of 65 °C and the heat resistance up to 80 °C, meaning that more energy-intensive heating is required [41,42]. The optimization experiments in this study determined a pH and temperature optima of 7.0 and 70 °C, respectively, which was slightly above the declared values. SEB Pro FL100 was sourced because of its declared maximum activity of 100,000 HUT/g at acidic pH of 3–5, and a comparatively low-temperature optimum of 40–55 °C (manufacturer’s specifications). These conditions would be attractive for an industrial-scale chitin extraction process because deproteination could be performed immediately following acid demineralization, with minimal pH adjustment. A lower process temperature is advantageous, as it results in energy savings. In this study, optimization experiments resulted in a theoretical residual protein content of 6.18%. The performance was best at lower pH, but activity was highest near the upper-temperature limit.

## 3. Materials and Methods

### 3.1. Reagents

Two different proteases, SEB Pro FL100 and Sea-B Zyme L200, were purchased from Enzyme Innovation (Chino, CA, USA). Analytical-grade sodium hydroxide and hydrochloric acid were purchased from Fisher Scientific (Ottawa, ON, Canada) and used in 10 wt% solutions for pH adjustment. Amino acid standards were purchased from Sigma-Aldrich (St. Louis, MO, USA).

### 3.2. Preparation of Shell and Demineralization

Fresh shrimp shells (*Pandalus Borealis*) were collected from a shrimp processing plant in Newfoundland, Canada. The shells were chilled on ice, transported to the laboratory, and further processed on the same day. Shells were subjected to a pre-treatment and demineralization process performed in a pilot-scale system. During pre-treatment, 319 kg of shrimp shell was ground, washed, and pressed, resulting in 123.6 kg of pressed wet shell with a homogeneous particle size of 1.4 mm. The weight reduction observed during pre-treatment (wet basis) was 61.27%. Demineralization was achieved by mixing 57 kg of the pre-treated shell with water to a reaction volume of 400 L. Demineralization was achieved within 3 h at 20 °C under continuous mixing by the stepwise addition of 16.39 L of 20° Bé (32%) hydrochloric acid, corresponding to a concentration of 4.09%. Weight reduction during demineralization was 36.67%. The resulting 36.1 kg of the pressed shell was frozen in portions for use in laboratory-scale deproteination experiments. The moisture, ash, and residual protein contents were determined as described below.

### 3.3. Analytical Methods

Ash and moisture contents were determined according to standard dry ashing and moisture procedures (AOAC 938.08 and 930.15). The residual protein content was estimated from the nitrogen content determined using the standard Kjeldahl method AOAC 954.01/988.05 (AOAC, 2012). Because both the chitin and proteins contain nitrogen, a common approach reported in the literature is to determine the total nitrogen content in a sample, as well as the nitrogen stemming from chitin, to calculate the residual protein content [36,40,41]. Varying data for chitin and chitosan nitrogen concentrations can be found in the literature. Theoretically, fully acetylated and deacetylated chitins contain 6.896 and 5.1% of nitrogen, respectively [27,42]. The natural nitrogen content of crustaceans is species-dependent, and varies between 6–7% [18].

### 3.4. Caustic Deproteination

A “pure” chitin standard was produced by mixing 100 g of demineralized shell (78.48% moisture) in 7% NaOH solution to a total reaction volume of 1 L (1:10 shell-to-liquid ratio) and incubating the mixture under constant stirring at 70 °C for 2 h. For the calculation, it was assumed that all protein was completely removed from the shell, remaining 100% chitin (acetylated). As a result, it was assumed that all nitrogen in this sample stemmed from chitin (N_Chitin_). Samples produced by enzymatic deproteination (described below) were compared to this “pure” standard. The total nitrogen content was determined by Kjeldahl analysis, and the residual protein content was calculated using the following formula:

Res.Prot_Enz.DP_ = (N_Enz.DP_ − N_Chem.DP_) × 6.25 (1)

Where Res.Prot, residual protein; Enz.DP, enzymatic deproteination; Chem.DP, chemical deproteination; and 6.25, average nitrogen content in protein.

### 3.5. Gas Chromatography–Mass Spectrometer (GC-MS) Analysis

In addition to the Kjeldahl protein determination, the residual protein content of samples from the selected samples was also determined via gas chromatography (GC)–mass spectrometer (MS) (Thermo Fisher Trace 1300 GC/ISQ-LT MS) amino acid analysis, which is recognized as the method of highest precision for protein determination [20]. All amino acids were weighed separately and prepared in 0.1 N HCl to prepare a stock solution at a concentration of 0.5 mg/mL. Five different stock solution concentrations were used to build a calibration curve. The DL-Norleucine was used as an internal standard, at a 0.5 mg/mL concentration. The internal standard was added to both standards and samples. Both standards and samples were subjected to derivatization using N-tertbutyldimethylsilyl-N-methyltrifluoroacetamide (MTBSTFA). During the derivatization process, 50 µL aliquots of standards/samples were taken in a 10 mL test tube, and then dried at 70 °C under nitrogen for 5 min. After drying, 100 µL of neat MTBSTFA was added, followed by adding 100 µL of acetonitrile. The test tubes were tightly capped and were heated at 100 °C for 2 hours. The sample was then allowed to cool at room temperature, and 200 µL of acetonitrile was added again to the tubes. The samples were then transferred into GC vials for analysis. The GC analysis was carried out using an SLB-5ms, 20 m × 0.18 mm column with an internal diameter of 0.18 µm. The inlet temperature was 280 °C and was operated at the splitless mode. The split flow was maintained at 100 mL/min, and the splitless time was 0.3 min. The column flow was 0.5 mL/min. The GC oven was maintained at 60 °C for 0 min and ramped up to 100 °C at 20 °C/min; held for 1 min and ramped up to 290 °C at 10 °C/min; and held for 3 min and ramped up to 340 °C, then held for 2 min. The MS transfer line temperature was 320 °C, and the ion source temperature was 280 °C. The standards and samples were scanned using MS in the range of 40–639 (*m/z*).

### 3.6. Enzymatic Deproteination

Deproteination experiments were carried out in 2500 mL beakers placed in an OLS200 water bath (Grant Instruments, Cambridge, UK). Agitation was provided by a EUROSTAR 60 overhead stirrer (IKA Works Inc., Wilmington, NC, USA) with an AN R1382 propeller stirrer, which was 3-bladed (IKA Works Inc., NC, USA). The pH was monitored using an HQ40D portable multi-meter (HACH, London, ON, Canada). In each experiment, 200 g of pre-treated and demineralized shell (described above) was mixed with pre-heated distilled water to a total reaction volume of 2000 mL before the enzyme addition. The pH was adjusted to the specified level, with 10% HCl and NaOH solutions. The solution was stirred for at least 30 min, or until the reaction temperature was reached and the pH remained steady. Then, the enzyme was added (Table 7) and the solution was stirred for the pre-determined reaction time. Upon completion, the shells were strained and washed for 5 min under running water before being hand-pressed and dried at 105 °C for 24 h.

### 3.7. Experimental Design and Statistical Analysis

A 2^4^ factorial design was created to investigate the factors of enzyme concentration, temperature, reaction time, and pH. A total of 16 different combinations were tested (1 replicate) in addition to 3 centre point runs for a total of 19 runs. Statistical evaluation was performed using Minitab software. High and low levels were set based on the manufacturer’s recommendations for the working range of the enzyme, which helps us to determine the optimal levels for desired results, compare enzyme efficiency, and understand how different enzyme concentrations impact the overall effectiveness. The high and low levels set for the two enzymes are summarized in Table 7.

An additional multifactorial design for SEB Pro FL100 was created to improve the prediction, with more narrow-level settings. A selection of 10 experiments were performed for the design, as shown in Table 8 and Table 9. Based on the initial 2^4^ factorial design results, all additional experiments were performed at a temperature of 55 °C. The reason for running additional experiments for SEB Pro FL100 is explained in the results section.

## 4. Conclusions

Protein removal from pre-treated and demineralized shrimp shells (*Pandalus borealis*) was optimized for two commercial proteases. The performance of papain (Sea B Zyme L200) was superior, leaving residual protein content of 2.01% (dry basis). The theoretic residual protein content after fungal protease treatment (SEB Pro FL100) was 6.18%, requiring a high enzyme concentration (>3%). In future studies, factors such as seasonal variability of the shell, crustacean species, and batch-to-batch differences should be examined, along with the physicochemical properties of chitin. To evaluate economic viability, a reduction of the enzyme concentration from 1.5–0.5% (Table 5, second best solution), as well as the reusability of the enzyme liquor for subsequent batches, should be assessed. Protein recovery from the liquid fraction should be further evaluated as an additional source of revenue.

## Figures and Tables

**Table 1:** 2^4^ Factorial design for SEB Pro FL100.

ID	pH	Temp (°C)	Conc (%)	Time (h)	Mean Total N (%)	SD Total N	Protein N (%)	Residual Protein (%)
1	6.00	55.0	0.10	12	8.95	0.30	2.23	13.92
2	6.00	40.0	4.00	2	8.83	0.00	2.11	13.21
3	6.00	55.0	4.00	12	8.93	0.16	2.21	13.79
4	6.00	40.0	0.10	12	9.26	0.06	2.54	15.87
5	3.00	55.0	0.10	2	8.47	0.09	1.75	10.94
6	3.00	40.0	4.00	12	7.81	0.05	1.09	6.82
7	3.00	55.0	4.00	2	7.74	0.05	1.02	6.37
8	3.00	55.0	4.00	12	7.52	0.16	0.80	5.03
9	6.00	40.0	0.10	2	9.31	0.02	2.59	16.22
10	6.00	55.0	0.10	2	9.10	0.00	2.38	14.85
11	4.50	47.5	2.05	7	7.89	0.09	1.17	7.31
12	3.00	55.0	0.10	12	8.58	0.04	1.86	11.62
13	3.00	40.0	0.10	12	8.52	0.06	1.80	11.28
14	6.00	40.0	4.00	12	8.77	0.11	2.05	12.83
15	3.00	40.0	4.00	2	8.08	0.02	1.36	8.49
16	4.50	47.5	2.05	7	7.87	0.03	1.15	7.17
17	6.00	55.0	4.00	2	8.82	0.11	2.10	13.14
18	4.50	47.5	2.05	7	7.82	0.01	1.10	6.87
19	3.00	40.0	0.10	2	8.83	0.08	2.11	13.18

**Table 2:** Additional multifactorial design experiments performed for SEB Pro FL100 with improved level settings.

ID	Time (h)	Conc (%)	pH	Mean Total N (%)	SD N	Protein N (%)	Residual Protein (%)
1	8	4	4.2	7.54	0.06	0.82	5.15
2	8	2	4.7	8.32	0.02	1.60	9.98
3	1	4	4.7	8.16	0.01	1.44	8.97
4	1	2	4.2	8.09	0.03	1.37	8.54
5	2	1	4.7	8.72	0.06	2.00	12.52
6	4	1	4.2	8.46	0.02	1.74	10.90
7	2	2	4.7	8.39	0.00	1.67	10.42
8	4	4	4.7	8.05	0.04	1.33	8.28
9	2	4	4.2	7.77	0.02	1.05	6.58
10	1	1	4.7	8.73	0.05	2.01	12.56

**Table 3:** Five best alternative solutions for SEB Pro FL100 deproteination.

Enzyme Concentration	pH	Residual Protein Content
2	4.2	8.23
4	4.7	8.31
2	4.7	10.36
1	4.2	10.58
1	4.7	12.70

**Table 4:** Kjeldahl analytical results of 24 factorial design for Sea-B-Zyme L200.

ID	pH	Temperature	Conc	Time	Mean Total N (%)	SD Total N	Protein N	Residual Protein (%)
1	7.00	70	0.50	2	7.31	0.03	0.59	3.68
2	7.00	60	1.50	8	7.19	0.02	0.47	2.95
3	7.00	60	1.50	2	7.32	0.04	0.60	3.75
4	7.00	60	0.50	8	7.37	0.06	0.65	4.04
5	5.50	60	0.50	2	8.09	0.01	1.37	8.59
6	6.25	65	1.00	5	7.44	0.00	0.72	4.51
7	5.50	70	1.50	2	7.53	0.04	0.81	5.07
8	5.50	60	0.50	8	7.87	0.06	1.15	7.19
9	5.50	70	0.50	8	7.53	0.05	0.81	5.03
10	5.50	60	1.50	2	7.75	0.01	1.03	6.47
11	7.00	60	0.50	2	7.60	0.01	0.88	5.47
12	7.00	70	1.50	8	7.22	0.04	0.50	3.10
13	5.50	70	0.50	2	7.85	0.10	1.13	7.09
14	6.25	65	1.00	5	7.38	0.04	0.66	4.13
15	5.50	70	1.50	8	7.73	0.02	1.01	6.33
16	7.00	70	1.50	2	7.05	0.57	0.33	2.06
17	6.25	65	1.00	5	7.48	0.03	0.76	4.72
18	5.50	60	1.50	8	7.58	0.07	0.86	5.36
19	7.00	70	0.50	8	7.20	0.05	0.48	3.03

**Table 5:** Sea-B-Zyme L200: Top five alternative solutions.

pH	Temp (°C)	Enzyme Concentration (%)	Reaction Time (h)	Predicted Residual Protein Content (%)
7	70	0.5	8	2.77
7	60	1.5	8	2.89
7	70	1.5	8	2.94
7	60	1.5	2	3.34
7	70	0.5	2	3.61

**Table 6:** Residual protein content in confirmation runs performed at optimal conditions as determined by the factorial design experiment: pH 7, 70 °C, and 1.5% Sea B Zyme for 2 h.

	Residual protein (%)	
	Kjeldahl Approach	Amino Acid Analysis
Sample ID	Mean ±SD (%)	Mean ± SD (%)
Before deproteination	17.28 ± 0.04	17.38 ± 0.18
1	2.25 ± 0.08	2.24 ± 0.14
2	0.00 ± 0.00 *	2.50 ± 0.10
3	1.40 ± 0.13	4.65 ± 0.25 *
4	0.93 ± 0.70	2.73 ± 0.58
5	5.43 ± 1.14 *	2.77 ± 1.14
6	1.56 ± 0.26	2.39 ± 0.11
7	0.75 ± 0.61	1.79 ± 0.12
8	0.84 ± 0.13	1.47 ± 0.01
9	2.87 ± 0.08	1.67 ± 0.17
10	2.50 ± 2.20	1.49 ± 0.22
11	1.09 ± 0.57	1.69 ± 0.03
12	1.59 ± 0.13	1.85 ± 0.08
13	1.18 ± 0.35	1.76 ± 0.20
14	2.37 ± 1.32	2.86 ± 0.76
15	1.87 ± 0.70	2.05 ± 1.06
16	2.68 ± 0.70	1.55 ± 0.50
17	1.09 ± 0.22	1.61 ± 0.11
18	1.56 ± 0.97	2.46 ± 0.26
19	1.78 ± 0.39	1.53 ± 0.15
20	1.31 ± 0.08	1.83 ± 0.37
Grand average	1.65 ± 0.53	2.01 ± 0.34

* Excluded from average and SD calculation.

**Table 7:** High and low levels for Sea-B-Zyme L200 and SEB Pro Fl100.

Factor	Sea-B-Zyme L200		SEB Pro FL100	
	Low	High	Low	High
pH	5.5	7	3	6
Temperature (°C)	60	70	40	55
Enzyme concentration (% of reaction volume)	0.5	1.5	0.1	4
Reaction time (h)	2	8	2	12

**Table 8:** SEB Pro FL100: multilevel factorial design, factor information.

Factor	Levels	Values
Reaction time (h)	4	1, 2, 4, and 8
Enzyme concentration (% of reaction volume)	3	1, 2, and 4
pH	2	4.2 and 4.7

**Table 9:** Additional experiments. Multilevel factorial design for SEB Pro FL100.

ID	Time	Enzyme Concentration	pH
1	8	4	4.2
2	8	2	4.7
3	1	4	4.7
4	1	2	4.2
5	2	1	4.7
6	4	1	4.2
7	2	2	4.7
8	4	4	4.7
9	2	4	4.2
10	1	1	4.7

## Data Availability

Available upon request.
